# Gene Variant in the NF-*κ*B Pathway Inhibitor *NFKBIA* Distinguishes Patients with Psoriatic Arthritis within the Spectrum of Psoriatic Disease

**DOI:** 10.1155/2019/1030256

**Published:** 2019-11-11

**Authors:** Pablo Coto-Segura, Eliecer Coto, Leire González-Lara, Belén Alonso, Juan Gómez, Elías Cuesta-Llavona, Rubén Queiro

**Affiliations:** ^1^Dermatology Division, Hospital Alvarez Buylla-Mieres, Mieres, Spain; ^2^Instituto Investigación Sanitaria del Principado de Asturias (IISPA), Oviedo, Spain; ^3^Department of Medicine, University of Oviedo, Oviedo, Spain; ^4^Molecular Genetics Unit, Hospital Universitario Central Asturias, Oviedo, Spain; ^5^Rheumatology Division, Hospital Universitario Central Asturias, Oviedo, Spain

## Abstract

**Background and Aims:**

The NF-*κ*B pathway has been implicated in the genetic aetiology of psoriatic disease. However, since most patients with arthritis have psoriasis, discerning the genetic contributions to both aspects of psoriatic disease is not easy. Our aim was to study the association of common polymorphisms in genes of the NF-*κ*B pathway in patients with psoriatic disease in order to dissect the contribution of this pathway in the appearance of each component (skin and joint) of the disease.

**Patients and Methods:**

We investigated the association between three common variants in *NFKB1* (rs230526), *NFKBIA* (rs7152376), and *NFKBIZ* (rs3217713 indel) and the risk of developing psoriatic disease. We genotyped a total of 690 psoriatic disease patients and 550 controls. Patients with cutaneous psoriasis of at least 10 years of evolution without associated arthritis were defined to have pure cutaneous psoriasis (PCP).

**Results:**

The rare *NFKBIA* rs7152376 C was significantly more frequent in the PsA group vs. controls (OR = 2.03 (1.3–3.1), *p* < 0.01). The difference was even higher between PsA and PCP patients (OR = 3.2 (2.1–5.1), *p* < 0.001). Neither *NFKB1* rs230526 nor *NFKBIZ* rs3217713 indel was associated with the risk of developing psoriatic disease as a whole compared to controls.

**Conclusions:**

Our study supports a significant effect of the *NFKBIA* gene on the risk of developing PsA, thus contributing to better discerning of the polymorphisms of this pathway that explain this risk within the spectrum of psoriatic disease. Additional studies with larger cohorts and from different populations are necessary to validate these results.

## 1. Introduction

In recent years, great advances have been made in the knowledge on the genetic basis of psoriasis and psoriatic arthritis (PsA). The nuclear factor-kappa beta (NF-*κ*B) is pivotal in the regulation of several biological processes, and its deregulation would affect immunological pathways that have been implicated in several pathological processes [1–3]. The NF-*κ*B pathway would play an important role in psoriasis [4–8]. Psoriatic skin exhibits increased expression of NF-*κ*B that would promote the expression of cytokines/chemokines that drive the proliferation of several immune cell types [6]. In this way, the NF-*κ*B is regarded as a mediator between immunity and the keratinocyte alteration characteristic of psoriasis [9]. This role is also supported by the fact that the blockade of the NF-*κ*B binding sites would reduce the expression of several proinflammatory cytokines implicated in psoriasis [8].

The transcriptional activity of NF-*κ*B is regulated by several cytoplasmic inhibitors (IkB*α*) that bind to the complex and prevent its translocation to the nucleus. Also, atypical nuclear proteins bind to NF-*κ*B and regulate its function. Among these nuclear inhibitors, I*κ*B*ζ* (encoded by *NFKBIZ*) would play a prominent role in the pathogenesis of psoriasis [7, 10]. I*κ*B*ζ* is induced by several proinflammatory molecules. In particular, its expression is regulated by IL-17A and might contribute to the activation of Th17-mediated pathways [11–13]. Therefore, I*κ*B*ζ* plays an important role in the pathogenesis of psoriatic disease through IL-17-mediated mechanisms [13, 14].

The HLA-Cw∗06 is the most recognised genetic risk factor for psoriasis. In addition, other genes that encode components of immune pathways have been linked to the risk for psoriasis and PsA. These include components of the NF-*κ*B pathway [15–18]. The association with NF-*κ*B polymorphisms has been investigated in cancer and several immunological diseases, including psoriasis and arthritis. The best characterised variant is rs28362491, a four-nucleotide biallelic indel in the *NFKB1* promoter that was linked to differences in gene expression and seems to be associated with the risk of developing several types of cancer [19, 20]. Recently, genome-wide association studies (GWAS) have found a significant association between psoriasis and single-nucleotide polymorphisms (SNPs) within *NFKB1*, *NFKBIA*, and *NFKBIZ* [15–17, 21]. Recently, we reported the association between psoriasis and an *NFKBIZ* intronic indel (rs3217713), conditioned by the Cw6 status, with the insertion allele significantly more frequent in Cw6-positive patients compared to Cw6-negative patients [22].

The greatest challenge in the study of the genetic aetiology of psoriatic disease is to dissect the genetic restriction elements of the cutaneous disease from those that would identify joint disease. The aim of our study herein was to characterise the genetic epidemiology of common NF-*κ*B gene polymorphisms and their association with the risk of developing psoriasis and PsA.

## 2. Patients and Methods

### 2.1. Study Population

The detailed information of the studied subjects is summarised in Table 1. All the participants were Caucasians from the region of Asturias (Northern Spain; total population: 1 million), older than 18 years. The study involved 690 patients with psoriatic disease (mean age: 48 ± 15 years; 55% men), recruited through the Dermatology and Rheumatology Department of Hospital Universitario Central de Asturias and Hospital Alvarez-Buylla, Mieres. They were diagnosed based on clinical findings and defined to have severe or nonsevere psoriasis according to the Psoriasis Area and Severity Index (PASI; severe when a PASI score ≥10). Early-onset psoriasis was defined as the onset of disease manifestations at age <40 years. The existence of arthritis was assessed by a rheumatologist according to the CASPAR (ClASsification of Psoriatic ARthritis) criteria. Accordingly, a total of 187 patients (27%) were diagnosed with PsA. Individuals affected with psoriasis for 10 or more years without developing arthritis were classified as pure cutaneous psoriasis (PCP; *n* = 309) cases. The 10-year period is the average time between the onset of psoriasis and the onset of arthritis. Thus, patients with psoriasis duration of this magnitude will most likely not develop arthritis [23]. Therefore, the population of PCP is a good comparator in relation to those patients with PsA.

The control group consisted of 550 nonrelated healthy individuals (mean age: 55 ± 16 years; 57% men) recruited through the Primary Healthcare Centres of Asturias, Spain. None of these controls had been diagnosed with psoriasis or arthritis at the time of inclusion in the study. There was no family history of psoriasis/PsA in the controls.

This study was approved by the Ethics Committee of Clinical Investigation of Principado de Asturias, and all the participants gave their written informed consent. The patient's cohort was registered as a Biobank Collection by Spanish Instituto de Salud Carlos III (reference C.0003441).

### 2.2. NF-*κ*B Variant Genotyping

We genotyped three variants in the *NFKB1* (rs230526), *NFKBIA* (rs7152376), and *NFKBIZ* (rs3217713 indel) genes. They were either previously directly associated or in strong linkage disequilibrium (LD) with other variants associated with cancer, psoriasis, arthritis, or coronary artery disease. Information on these variants including the flanking sequence, reported population frequencies, and LD values was obtained from the Ensembl website (http://www.ensembl.org) and LDlink (https://ldlink.nci.nih.gov/).

The DNA was obtained from 5 mL of blood. All the variants were genotyped through polymerase chain reaction (PCR) amplification of genomic DNA with specific primer pairs (Supplementary [Supplementary-material supplementary-material-1]) followed by digestion with a restriction enzyme (PCR-RFLP) and electrophoresis on agarose gels to visualise different alleles. In brief, approximately 100 ng of DNA was amplified (32 cycles of 95°C-30 s, annealing at 65°C-60 s, and 72°C-60 s) in a final volume of 30 *μ*l containing 10 pmol of each primer, 1 unit of Taq DNA polymerase, and 1X buffer. The amplifications were digested with 10 units of the appropriated restriction enzyme and electrophoresed on 4% agarose gels to visualise the alleles in a UV transilluminator.

The *NFKB1* rs230526 A/G was in complete LD (*D*′ = 1, *r*^2^ = 1) with rs28362491 (−94 delATTG), a common insertion/deletion (indel) polymorphism in the promoter region of *NFKB1* that has been widely studied in cancer and immune-mediated processes and was associated with differences in gene expression (the deletion would drive less promoter activity) [19]. *NFKBIA* rs7152376 was in complete LD with rs12883343 (*D*′ = 1.0, *r*^2^ = 1.0), an SNP previously associated with psoriasis and PsA in GWAS [17]. The *NFKBIZ* rs3217713 is a 23 nt insertion/deletion (indel) polymorphism that was previously associated with the risk of psoriasis [22]. The PCRs were electrophoresed on agarose gels to visualise the two indel alleles.

All patients were genotyped for *HLA-Cw6* (*PSORS1*).

### 2.3. Statistical Analysis

All patients and controls' anthropometric, analytical, and genetic data were stored in an Excel file. The genotype frequencies for each polymorphism were tested online for the Hardy–Weinberg equilibrium (http://www.oege.org/software/hwe-mr-calc.shtml). The statistical analysis was performed with R software (http://www.r-project.org). A logistic regression was used to compare the frequencies between the groups. Odds ratios (ORs) and 95% confidence intervals (95% CIs) were also calculated. A multivariate logistic regression was performed to determine the independent effect of the genotypes and the early onset, disease severity (PASI >10), and sex. *p* values <0.05 were considered statistically significant.

## 3. Results

We investigated the association between three common variants in *NFKB1* (rs230526), *NFKBIA* (rs7152376), and *NFKBIZ* (rs3217713 indel) and the risk of developing psoriasis/PsA or their main clinical outcomes. A total of 690 psoriatic disease patients, 187 (27%) with arthritis, and 550 controls were genotyped. The main characteristics of the study cohorts are summarised in Table 1.

The genotype frequencies for the three variants are summarised in Table 2. The minor allele frequencies (MAFs) in our population were almost identical to those reported for other populations of European ancestry. Allele and genotype frequencies did not significantly differ (*p* > 0.05) between patients and controls for the three gene variants, and thus, we concluded that none of these polymorphisms contributed significantly to the risk for psoriatic disease in our population. None of the three variants was associated with psoriatic disease after multiple logistic regression including sex and age as covariates.

We compared allele and genotype frequencies between controls and patients with PsA or PCP (Table 2). The rare *NFKBIA* rs7152376 C was significantly more frequent in the PsA group vs. controls (0.42 vs. 0.36; OR = 2.03 (1.3–3.1), *p* < 0.01). This allele was significantly less frequent in patients with PCP compared to controls (0.31 vs. 0.42; *p* < 0.05). Compared to PCP patients, PsA patients showed a significantly higher frequency of rs7152376 C (0.42 vs. 0.31; OR = 3.2 (2.1–5.1), *p* < 0.001). In reference to the genotypes, carriers of rs7152376 C (CC + CT) were more frequent in PsA patients compared to controls (0.66 vs. 0.58; *p*=0.06). The frequency of these carriers was significantly higher in PsA vs. PCP groups (*p*=0.004).

Early-onset psoriasis, female sex, and Cw6 negative were significantly more frequent in PsA patients compared to PCP patients (Table 2). We performed a multiple logistic regression including early onset, sex, Cw6, and *NFKBIA* rs7152376 genotype (CC + CT vs. TT), and the four variables remained significantly associated with PsA vs. PCP (Table 3).

## 4. Discussion

Several published papers have identified genetic factors that might differentiate psoriasis and psoriatic arthritis (PsA) [17, 24, 25]. We performed a genetic association study of psoriatic disease, PCP, and PsA and three common variants in key components of the NF-*κ*B pathway. None of these polymorphisms was significantly associated with the risk of developing psoriatic disease as a whole in our population. However, we found a significant association between the *NFKBIA* variant and PsA.

*NFKB1* encodes a protein p105 that is further processed to the p50 protein. Previous studies have reported significant associations between common *NFKB1* variants and psoriasis, including large-scale genome analysis involving thousands of patients and controls [26]. Most of the studies based on cohorts of limited size studied the *NFKB1* −94 ins/delATTG polymorphism and found low significant associations. The deletion allele was initially associated with an increased risk for ulcerative colitis and would be linked to reduced expression of *NFKB1* compared to the insertion allele [19]. We did not study this variant; instead, we genotyped *NFKB1* rs230526 that was in complete LD with the promoter indel and thus served as a surrogate marker for the functional promoter variant. In reference to the main clinical outcomes, at least one *NFKB1* variant has been associated with psoriasis severity [27]. To our knowledge, this SNP has not been previously investigated in other diseases, although it was in high LD with the common rs28362491 (−94 ATTG indel) in the *NFKB1* promoter that was associated with differences in gene expression (the deletion would drive less promoter activity) and has been widely studied in cancer and immune-mediated processes [19, 20, 28], including the risk for type 2 diabetes in our population [29]. The promoter indel was also associated with psoriasis in a case-control study of 519 Chinese psoriasis vulgaris patients and 541 matched controls (*p*=0.031), but the difference was not still significant after correction for multiple comparisons [30].

In our study, the *NFKB1* frequencies did not differ between total psoriatic disease and controls, or the severity, onset age, or disease severity. This variant did not differ between the PsA and PCP groups.

We studied an *NFKBIA* variant (rs7152376 C/T) that was in complete LD with an SNP (rs12883343 C/G) previously associated with PsA in a meta-analysis of 6 GWAS [17]. The rare G-allele frequency was significantly increased in psoriasis patients vs. controls (0.45 vs. 0.41; meta-OR = 1.16). In the same study, the rare allele was also significantly increased in PsA patients compared to controls (0.46 vs. 0.41; meta-OR = 1.22). The association between this *NFKBIA* SNP and PsA was recently confirmed by a case-control study in a Chinese cohort [31]. Interestingly, in our study, the rare rs7152376 allele was a disease marker for PsA, with a maximum difference between PsA and PCP patients (*p* < 0.001). These results provide insights into the pathogenetic differences between skin psoriasis and PsA and pointed to *NFKBIA* as an important determinant of the risk of developing joint disease within the spectrum of psoriatic disease.

Psoriatic disease is associated with high cardiovascular comorbidity. Currently, it is assumed that the link between the two processes is the inflammation itself. We have recently demonstrated that NFKB1 variation was an independent risk factor for developing type 2 diabetes, while the NFKBIZ variant was an independent risk factor for developing early-onset coronary artery disease [29, 32]. Taken together, our data suggest that genetic alterations in the NF-*κ*B pathway play an important role in the pathogenesis of psoriatic disease and its comorbidities, establishing a common link for all these manifestations (arthritis, cardiovascular risk factors, and cardiovascular adverse events).

Our study has several limitations, mainly the limited sample size and the fact that it was based on a single population. In addition, the *NFKBIA* variants associated with PsA were intronic, and a functional effect on gene expression and/or protein function has not been established. Most likely, these SNPs are in linkage disequilibrium with other variants that have a functional effect and are responsible for the observed genetic associations. Studies focused to characterise these *NFKBIA* variants are of upmost relevance, as well as functional studies to define the functional differences between the alleles. However, our results support a differential risk in this pathogenic pathway that may help to discern which patients with psoriatic disease have a higher risk of developing PsA.

## 5. Conclusions

Our study supports a significant effect of the *NFKBIA* gene (a key component of the NF-*κ*B pathway) on the risk of developing PsA. Additional studies with larger cohorts and from different populations are necessary to confirm these findings.

## Figures and Tables

**Table 1:** Main characteristics of the total group and different psoriatic disease groups.

	Total PD, *N* = 690	No PsA, *N* = 503	PsA, *N* = 187	PCP, *N* = 309	*p* value
Onset age (mean, years)	27	27	28	21	
Early onset	176 (26%)	135 (27%)	41 (22%)	36 (12%)	0.002
PASI >10	371 (54%)	249 (50%)	122 (65%)	190 (61%)	0.40
Male sex	381 (55%)	292 (58%)	89 (48%)	182 (59%)	0.01
Cw6 positive	304 (44%)	234 (47%)	70 (37%)	166 (54%)	<0.001
*NFKB1* rs230526 A	42%	42%	41%	40%	0.76
*NFKBIA* rs7152376 T	35%	33%	42%	31%	<0.001
*NFKBIZ* rs3217713 ins	20%	19%	20%	17%	0.23

*p* values correspond to the PsA vs. PCP groups. For the three *NFKB* polymorphisms, we show the minor allele frequencies. PD: psoriatic disease; PsA: psoriatic arthritis; PCP: pure cutaneous psoriasis (absence of arthritis after 10 or more years of psoriasis onset).

**Table 2:** Genotype and allele frequencies in different psoriatic disease groups and controls.

	Controls, *N* = 550	Total PD, *N* = 690	PsA, *N* = 187	PCP, *N* = 309	*p* value, OR (95% CI)
*NFKB1 rs230526 A/G*					AA vs. AG + GG
AA	77 (0.14)	108 (0.16)	30 (0.16)	42 (0.14)	*p*=0.45
AG	264 (0.48)	359 (0.52)	95 (0.51)	164 (0.53)	OR = 1.21
GG	209 (0.38)	223 (0.32)	62 (0.33)	103 (0.33)	0.73–2.02

*NFKBIA rs7152376 T/C*					CC + CT vs. TT
TT	231 (0.42)	291 (0.42)	64 (0.34)	147 (0.48)	*p*=0.004
TC	242 (0.44)	314 (0.46)	89 (0.48)	133 (0.43)	OR = 1.74
CC	77 (0.14)	85 (0.12)	34 (0.18)	29 (0.09)	1.20–2.54

*NFKBIZ rs3217713 indel*					Del/Del vs. Ins/Ins + Ins/Del
INS/INS	336 (0.61)	451 (0.65)	124 (0.66)	210 (0.68)	*p*=0.06
INS/DEL	193 (80.35)	207 (0.30)	51 (0.27)	90 (0.29)	OR = 2.29
DEL/DEL	21 (0.04)	32 (0.05)	12 (0.06)	9 (0.03)	0.94–5.53

*p* and OR values correspond to the putative PsA risk vs. non‐risk genotypes. PD: psoriatic disease; PsA: psoriatic arthritis; PCP: pure cutaneous psoriasis (absence of arthritis after 10 or more years of psoriasis onset); OR: odds ratio; CI: confidence interval.

**Table 3:** Logistic regression for psoriatic arthritis (PsA) vs. pure cutaneous psoriasis (PCP), for the variables significantly different between the two groups.

	*p* value (univariate)	*p* value (multivariate)
Cw6	0.0004	0.005
Early onset	0.003	0.002
Sex	0.015	0.016
NFKBIA rs7152376 C carriers	0.004	0.001

*p* values correspond to the univariate and the multivariate (linear generalised model) analysis.

## Data Availability

The patient's cohort was registered as a Biobank Collection by Spanish Instituto de Salud Carlos III (reference C.0003441). The datasets used and analysed during the current study are available from the corresponding author on reasonable request.

## Abbreviations

PsA: Psoriatic arthritis
NF-*κ*B: Nuclear factor-kappa beta
IkB*α*: Inhibitor of nuclear factor-kappa B subunit alpha
I*κ*B*ζ*: Inhibitor of nuclear factor-kappa B subunit *ζ*
IL-17A: Interleukin-17A
HLA-Cw∗06: Human leukocyte antigen-Cw∗06
NFKB1: Nuclear factor-kappa beta 1
GWAS: Genome-wide association studies
NFKBIA: Nuclear factor-kappa beta IA
NFKBIZ: Nuclear factor-kappa beta IZ
PASI: Psoriasis Area and Severity Index
CASPAR: ClASsification of Psoriatic ARthritis
PCP: Pure cutaneous psoriasis
LD: Linkage disequilibrium
DNA: Deoxyribonucleic acid
PCR: Polymerase chain reaction
RFLP: Restriction fragment length polymorphism
PSORS1: Psoriasis susceptibility gene 1
OR: Odds ratio
CI: Confidence interval
MAFs: Minor allele frequencies.

## Supplementary Materials

Supplementary Materials Supplementary Table 1: primers and PCR conditions for genotyping the three NFKB pathway gene variants. The NFKB1 and NFKBIA variants were genotyped through polymerase chain reaction (PCR) amplification of genomic DNA with specific primer pairs followed by digestion with a restriction enzyme (PCR-RFLP) and electrophoresis on agarose gels to visualise different alleles. The NFKBIZ rs3217713 is a 23 nt insertion/deletion (indel) polymorphism that was previously associated with the risk of psoriasis. The PCRs were electrophoresed on agarose gels to visualise the two indel alleles.
