# Supplementary material for: Gene Variant in the NF-κB Pathway Inhibitor NFKBIA Distinguishes Patients with Psoriatic Arthritis within the Spectrum of Psoriatic Disease
Source: Biomed Res Int. 2019 Nov 11;2019:1030256. doi: 10.1155/2019/1030256 (PMC6877981; doi:10.1155/2019/1030256)
Supplement: Supplementary Materials — Supplementary Table 1: primers and PCR conditions for genotyping the three NFKB pathway gene variants. The NFKB1 and NFKBIA variants were genotyped through polymerase chain reaction (PCR) amplification of genomic DNA with specific primer pairs followed by digestion with a restriction enzyme (PCR-RFLP) and electrophoresis on agarose gels to visualise different alleles. The NFKBIZ rs3217713 is a 23 nt insertion/deletion (indel) polymorphism that was previously associated with the risk of psoriasis. The PCRs were electrophoresed on agarose gels to visualise the two indel alleles. [file 1030256.f1.pdf]

Supplementary table 1. Primers and PCR conditions for genotyping the three NFKB pathway gene variants.

The *NFKB1* and *NFKBIA* variants were genotyped through polymerase chain reaction (PCR) amplification of genomic DNA with specific primer-pairs followed by digestion with a restriction enzyme (PCR-RFLP) and electrophoresis on agarose gels to visualise the different alleles.

The *NFKBIZ* rs3217713 is a 23 nt insertion/deletion (indel) polymorphism that was previously associated with the risk of Psor**.** The PCRs were electrophoresed on agarose gels to visualise the two indel alleles.

**PCR-PRIMERS,**<br>**Forward / reverse**	**Restriction Enzyme**	**Allele size**<br>**Base pairs**
NFKB1 rs230526 A/G<br>5´ ATG TCT GTA GTC CCT GCT GAA CC<br>5´ GGA GCG AAG GCA ACC CGT AC	**TaqI**	**A: 670 bp**<br>**G: 390 + 280 bp**
NFKBIA rs7152376 A/G<br>5´ GGA GGT CAG GGT GCT CTT AGG<br>5´ AGG GAG ATA GGG GAC CTG CAG	***SmaI***	**A: 509 bp**<br>**G: 339 + 170 bp**
NFKBIZ rs3217713<br>5´ AGGATCGCAAAAGTGGCCGCACAG<br>5´ ATGAATTATCAGACATCAG GAAGGGGG	**None**	**Ins: 230 bp**<br>**Del: 207 bp**
